# Baseline Gastrointestinal Eosinophilia Is Common in Oral Immunotherapy Subjects With IgE-Mediated Peanut Allergy

**DOI:** 10.3389/fimmu.2018.02624

**Published:** 2018-11-22

**Authors:** Benjamin L. Wright, Nielsen Q. Fernandez-Becker, Neeraja Kambham, Natasha Purington, Dana Tupa, Wenming Zhang, Matthew A. Rank, Hirohito Kita, Kelly P. Shim, Bryan J. Bunning, Alfred D. Doyle, Elizabeth A. Jacobsen, Scott D. Boyd, Mindy Tsai, Holden Maecker, Monali Manohar, Stephen J. Galli, Kari C. Nadeau, R. Sharon Chinthrajah

**Affiliations:** ^1^Division of Allergy, Asthma, and Clinical Immunology, Department of Medicine, Mayo Clinic Arizona, Scottsdale, AZ, United States; ^2^Division of Pulmonology, Phoenix Children's Hospital, Phoenix, AZ, United States; ^3^Sean N. Parker Center for Allergy and Asthma Research, Stanford University School of Medicine, Stanford, CA, United States; ^4^Department of Pathology, Stanford University School of Medicine, Stanford, CA, United States; ^5^Division of Allergic Diseases, Internal Medicine, Mayo Clinic, Rochester, MN, United States; ^6^Department of Biochemistry and Molecular Biology, Mayo Clinic Arizona, Scottsdale, AZ, United States; ^7^Institute for Immunity, Transplantation, and Infection, Stanford University School of Medicine, Stanford, CA, United States; ^8^Department of Microbiology and Immunology, Stanford University School of Medicine, Stanford, CA, United States

**Keywords:** peanut food allergy, Eosinophilic Esophagitis, eosinophil, adverse event, biopsy, endoscopy, gastrointestinal, oral immunotherapy

## Abstract

**Rationale:** Oral immunotherapy (OIT) is an emerging treatment for food allergy. While desensitization is achieved in most subjects, many experience gastrointestinal symptoms and few develop eosinophilic gastrointestinal disease. It is unclear whether these subjects have subclinical gastrointestinal eosinophilia (GE) at baseline. We aimed to evaluate the presence of GE in subjects with food allergy before peanut OIT.

**Methods:** We performed baseline esophagogastroduodenoscopies on 21 adults before undergoing peanut OIT. Subjects completed a detailed gastrointestinal symptom questionnaire. Endoscopic findings were assessed using the Eosinophilic Esophagitis (EoE) Endoscopic Reference Score (EREFS) and biopsies were obtained from the esophagus, gastric antrum, and duodenum. Esophageal biopsies were evaluated using the EoE Histologic Scoring System. Immunohistochemical staining for eosinophil peroxidase (EPX) was also performed. Hematoxylin and eosin and EPX stains of each biopsy were assessed for eosinophil density and EPX/mm^2^ was quantified using automated image analysis.

**Results:** All subjects were asymptomatic. Pre-existing esophageal eosinophilia (>5 eosinophils per high-power field [eos/hpf]) was present in five participants (24%), three (14%) of whom had >15 eos/hpf associated with mild endoscopic findings (edema, linear furrowing, or rings; median EREFS = 0, IQR 0–0.25). Some subjects also demonstrated basal cell hyperplasia, dilated intercellular spaces, and lamina propria fibrosis. Increased eosinophils were noted in the gastric antrum (>12 eos/hpf) or duodenum (>26 eos/hpf) in 9 subjects (43%). EPX/mm^2^ correlated strongly with eosinophil counts (*r* = 0.71, *p* < 0.0001).

**Conclusions:** Pre-existing GE is common in adults with IgE-mediated peanut allergy. Eosinophilic inflammation (EI) in these subjects may be accompanied by mild endoscopic and histologic findings. Longitudinal data collection during OIT is ongoing.

## Introduction

Peanut allergy is a potentially fatal disease affecting 0.5–1% of the general population (1–3) with rates as high as 3% in young children (4). Possibly fueled by an era of widespread early peanut avoidance (5, 6), the disease has doubled over the past decade (7) and tends to persist through adulthood for a majority of individuals (8). The current standard of care for patients entails food avoidance and acute management of allergic reactions (9). Emerging evidence suggests that desensitization can be achieved by a method of graduated peanut administration termed oral immunotherapy (OIT) (10–12). Following an initial escalation phase, OIT subjects are desensitized to the offending food antigen during a series of gradual dose adjustments until a pre-specified maintenance dose is reached. Rates of clinical desensitization are excellent ranging from 80 to 90% for food allergy (10, 13, 14); however, the durability of this immunologic response remains in question. In most patients, continuation of “regular” therapy appears to be necessary in order to maintain desensitization (15, 16). However, a few trials have now shown that desensitization can be maintained for some time after a period of discontinuation of therapy (allergen avoidance) (12, 17–19). This outcome is termed “sustained unresponsiveness (SU),” and refers to patients who successfully pass an oral food challenge after a defined period of allergen avoidance following OIT (20).

Questions regarding safety are the principal obstacles to broader acceptance and availability of OIT. Specifically, 5–36% of subjects withdraw from clinical trials because they cannot tolerate the treatment (11, 21–23). Moreover, adverse events (AEs) are not limited to a small subset of individuals. A recent retrospective study of AEs during peanut OIT suggested 80% of subjects experience OIT-related AEs (22). Although serious AEs, such as anaphylaxis are rare, the frequency of OIT-related side effects has led some investigators and allergists to question whether this intervention is superior to avoidance (24). The most common AEs related to OIT discontinuation that have been reported are gastrointestinal symptoms, specifically abdominal pain (25). While OIT-induced gastrointestinal symptoms are more common during the buildup phase, they may occur at any point during therapy (26, 27).

Most concerning is the occurrence of eosinophilic gastrointestinal disease (EGID) in some subjects undergoing OIT (28). EGIDs are a group of diseases characterized by eosinophil-rich inflammation affecting different locations of the digestive tract: the esophagus, stomach, and intestine. Eosinophils can normally reside in the mucosa of the stomach and intestine, but are not normally found in the esophagus (29). Eosinophilic esophagitis (EoE) is the most common EGID and is typically a chronic immune disorder mediated by antigen exposure. EoE is defined by clinical and histopathological criteria, in the absence of other causes. Clinical symptoms may include the following: reflux-like symptoms, abdominal pain and/or vomiting that is refractory to reflux treatment, dysphagia and/or food impaction in conjunction with histological evidence of dense eosinophilic infiltration of the mucosa (≥15 eosinophils per high-power field [eos/hpf]) (30).

A meta-analysis (31) and a recent retrospective review (25) estimated the incidence of EoE during OIT at rates of 2.7 and 5.1%, respectively. Both are likely underestimates as most subjects with gastrointestinal symptoms do not routinely undergo upper endoscopy for diagnostic confirmation. Indeed, some centers estimate rates of OIT-induced EoE as high as 8–14% when rates of discontinuation due to gastrointestinal symptoms (i.e., abdominal pain or vomiting) are used as a surrogate measure (32). Notwithstanding, diagnosis of EoE based on symptoms alone is inherently limited. This phenomenon has sparked debate within the food allergy community given the potential risk of generating a chronic disease *de novo*. A confirmed diagnosis of EoE requires long-term treatment with a proton pump inhibitor (PPI), swallowed corticosteroids, or food avoidance despite time already spent in the desensitization process. Fortunately, resolution of EoE is observed in most patients with OIT-induced EoE on discontinuation of OIT (28).

In a prospective study evaluating OIT and the incidence of EGID, 8 of 128 participants undergoing OIT for milk and/or egg were diagnosed with EGID after 15–48 months of starting OIT (six with EoE and two with eosinophilic gastroenteritis). Interestingly, OIT was maintained in five of the six EoE patients while PPI therapy with or without swallowed steroids was initiated. One patient refused treatment with medications and thus, OIT was discontinued. In all six patients, symptoms resolved and in the three of five patients with repeat esophagogastroduodenoscopies (EGDs), there was histological remission (27).

Some have suggested that subjects with OIT-induced EoE may have pre-existing, subclinical disease. This notion is supported by estimates suggesting that the prevalence of EoE in patients with IgE-mediated food allergy (1:20) is 125 times more common than in the general population (1:2,500) (32). Currently, there is a critical need to determine whether food-allergic patients who are avoiding culprit allergens have underlying eosinophilic inflammation (EI) in the gastrointestinal tract and whether the presence of EI will affect their outcomes with OIT. Unfortunately, no biomarkers or minimally invasive measurement techniques have been validated to diagnose EoE or EGIDs without endoscopy (33). The objective of this study was to evaluate the presence of gastrointestinal eosinophilia (GE) in a small cohort of adult subjects with IgE-mediated peanut allergy prior to initiation of OIT.

## Methods

### Study population

Participants were recruited as part of a randomized, double-blind, placebo-controlled, phase II clinical trial studying peanut OIT at the Sean N. Parker Center for Allergy and Asthma Research at Stanford University from April 2014 to March 2016 (clinicaltrials.gov; NCT02103270) (34). All aspects of the studies from which data was obtained were authorized by the Stanford University School of Medicine Institutional Review Board (Stanford, CA). Peanut allergy was confirmed with clinical history, skin prick tests, and a positive challenge to peanut during a double-blind, placebo-controlled food challenge (DBPCFC). A clinical history of EGID was a key exclusion criterion. A subset of participants, aged >18 years old, was consented to participate in an IRB approved sub-study, with a separate data safety monitoring board, involving EGDs prior to starting OIT. A comprehensive gastrointestinal symptom questionnaire was given to participants to assess clinical symptoms (see Supplementary Material). Symptoms were assessed within 1 month of upper endoscopy. The gastrointestinal symptom questionnaire included a 1 month recall period and assessed symptoms of reflux, abdominal pain, constipation, poor appetite, dysphagia, time required for eating, food refusal, and vomiting. None of the subjects enrolled in this study had previously undergone an upper endoscopy.

### Esophagogastroduodenoscopies

All participants were consented for the research study and for the procedure. EGDs were performed under conscious sedation by trained gastroenterologists (N.F.B). Endoscopic biopsies were obtained from five sites: the proximal esophagus (PE), middle esophagus (ME), and distal esophagus (DE), as well as the gastric antrum and proximal duodenum. Four passes were performed at each location and one biopsy was obtained with each pass. Given the need to conserve samples for future mechanistic studies, only one biopsy from each site was analyzed by histology. Standardized reporting of endoscopic findings was undertaken, using the Eosinophilic Endoscopic Reference Score (EREFS) (35). The inflammatory and fibrostenotic scores were calculated as per Dellon et al. (36).

### Evaluation of gastrointestinal pathology

Sections from the PE, ME, DE, gastric antrum, and proximal duodenum were stained with hematoxylin and eosin (H + E). A gastrointestinal pathologist (N.K.), who was blinded to the clinical characteristics and demographic data of the individual participants, quantified peak eosinophils counts in a single hpf in an area of highest density and scored esophageal biopsy slides using the Eosinophilic Esophagitis Histologic Scoring System (EoEHSS), as previously described (37). Briefly, the EoEHSS not only quantifies EI but also informs as to the severity and extent of inflammation and histological abnormality, and consists of 8 features: eosinophilic inflammation (EI), basal zone hyperplasia (BZH), dilated intercellular spaces (DIS), lamina propria fibrosis (LPF), eosinophilic abscess (EA), eosinophil surface layering (SL), surface epithelial alteration (SEA), and dyskeratotic epithelial cells (DEC) (37). Each feature is scored separately for grade (severity) or stage (extent) of abnormality using a 4-point scale (0 = normal; 3 = most severe or extensive). These features were analyzed for each participant for the PE, ME, and DE biopsies. Greater than 5 eos/hpf in the esophagus was considered abnormal and any eosinophils above the published upper limits of normal in the stomach (>12 eos/hpf) or duodenum (>26 eos/hpf) were considered abnormal (29).

### Immunohistochemical (IHC) staining for eosinophil peroxidase (EPX)

Tissue sectioning and IHC staining was performed at the Pathology Research Core (Mayo Clinic, Rochester, MN) using the Leica Bond RX stainer (Leica). Formalin-fixed-paraffin-embedded (FFPE) tissues were sectioned at five microns and IHC staining was performed on-line. Slides for EPX stain were retrieved for 20 min using Epitope Retrieval 1 (Citrate; Leica) and incubated in Protein Block (Dako) for 5 min. The EPX primary monoclonal antibody [clone MM25-82.2 (38)] was diluted to 1:750 in Background Reducing Diluent (Dako) and incubated for 15 min.

The detection system used was the Polymer Refine Detection System (Leica). This system includes a hydrogen peroxidase block, post-primary and polymer reagent, DAB, and hematoxylin. Immunostaining visualization was achieved by incubating slides 10 min in DAB and DAB buffer (1:19 mixture) from the Bond Polymer Refine Detection System. To this point, slides were rinsed between steps with 1X Bond Wash Buffer (Leica). Slides were counterstained for 5 min using Schmidt hematoxylin (instead of the hematoxylin provided with the Refine kit) and molecular biology grade water (1:1 mixture), followed by several rinses in 1X Bond wash buffer and distilled water. Once the immunochemistry process was completed, slides were removed from the stainer and rinsed in tap water for 5 min. Slides were dehydrated in increasing concentrations of ethyl alcohol and cleared in 3 changes of xylene prior to permanent cover-slipping in xylene-based medium.

### Analysis of EPX stains

Tissue sections were digitized (Aperio AT Turbo, Leica Biosystems, Buffalo Grove, IL) and peak eosinophil counts (PEC) were evaluated using an area equivalent to 1 hpf (0.24 mm^2^). EPX deposition was quantified by an automated pixel algorithm with Aperio ImageScope software (version 11.2.0.780, Aperio Technologies, Vista, CA). Only pixels that stained strongly or moderately positive according to the algorithm were considered positive (see Supplementary Figure E1). The peak number of EPX positive pixels within 1 hpf was divided by the epithelial area (mm^2^) analyzed. Automated measurements of EPX/mm^2^ were made in tissue sections cut from each gastrointestinal biopsy. Manual counts of EPX positive nuclei were also performed in a single hpf in an area of highest density.

### Statistical analysis

Descriptive statistics were reported for baseline characteristics, EREF scores, eosinophil counts, EPX measurements, and EoEHSS scores. The inflammatory score was calculated by summing the exudate, edema, and furrows scores, and the fibrostenotic score was the sum of the rings and stricture scores. The total score was the sum of the inflammatory and fibrostenotic scores. Spearman's test was used to determine correlations between eos/hpf and EPX/mm^2^. Comparisons of eos/hpf by H + E and eos/hpf by EPX immunohistochemistry were performed using a Mann-Whitney test. All analyses were conducted using GraphPad Prism version 7.0f for Windows and R v3.4.3.

## Results

### Clinical characteristics

Twenty one adults, median age 27 years old, were enrolled in the study and underwent baseline EGDs. The majority of participants were Caucasian males. All participants had histories of peanut allergy with elevated peanut-specific IgE (median 16.81 kU/L) and a median peanut skin prick test of 12.5 mm as well as a confirmatory positive reaction on a DBPCFC. Overall, the cohort included allergic individuals with elevated median total IgE of 617.32 kU/L, median absolute eosinophil counts of 200 cells/uL, and other allergic comorbid conditions, such as asthma (81%), allergic rhinitis (86%), and a history of atopic dermatitis (52%). Forty-eight percent of adults had other food allergies in addition to being peanut allergic (Table 1; Supplementary Table E2). Details of other food allergies and concurrent medication use in regards to proton pump inhibitors (PPIs) and inhaled corticosteroids are in Supplementary Table E2. None of the participants were on concurrent PPI at the time of endoscopy.

**Table 1:** Baseline characteristics.

**Characteristic**	**Subjects (*n* = 21)**
Age at baseline (y), median (IQR)	26.5 (22.5–34.5)
Males (*n*, %)	16 (76)
White (*n*, %)	15 (71)
**Atopic conditions (*****n*****, %)**
Asthma	17 (81)
Allergic rhinitis	18 (86)
Atopic dermatitis	11 (52)
Other food allergies	10 (48)
Total IgE level (IU/L), median (IQR)^*^	617.32 (215.94, 1,125.45)
Peanut-specific IgE level (kU_A_/L), median (IQR)	16.81 (7.22, 166.90)
Peanut-specific IgG4 level (μg/mL), median (IQR)	0.31 (0.11, 0.43)
Peanut skin prick test (mm), median wheal size (IQR)	12.5 (7.5, 17.5)
Absolute eosinophil counts (cells/μL), median (IQR)	200 (112.5, 322.5)^*^

* *Missing for 1 subject*.

### Gastrointestinal symptoms

All patients were generally asymptomatic at the time of endoscopy. Questionnaires (Supplementary Material) were administered within 1 month of EGD. None of the participants reported a history of dysphagia. Five participants reported mild abdominal pain < 3 times per month and one subject reported mild reflux (not associated with food), < 3 times per month. Two of the five participants with reported abdominal pain and one participant with mild reflux had mild abnormalities on endoscopy. None of the participants with mild abdominal pain or reflux had any appreciable eosinophils in the PE, ME or DE by H + E staining, while others who were found to have any eosinophils in the esophagus (*n* = 6) did not report symptoms. Two participants with abdominal pain < 3 times per month had PEC of >26 in the duodenum by H + E staining; the remainder had eosinophils in the gastric antrum or duodenum, but less than noted thresholds (Supplementary Table E1).

### Endoscopic findings

**Figure 1:**
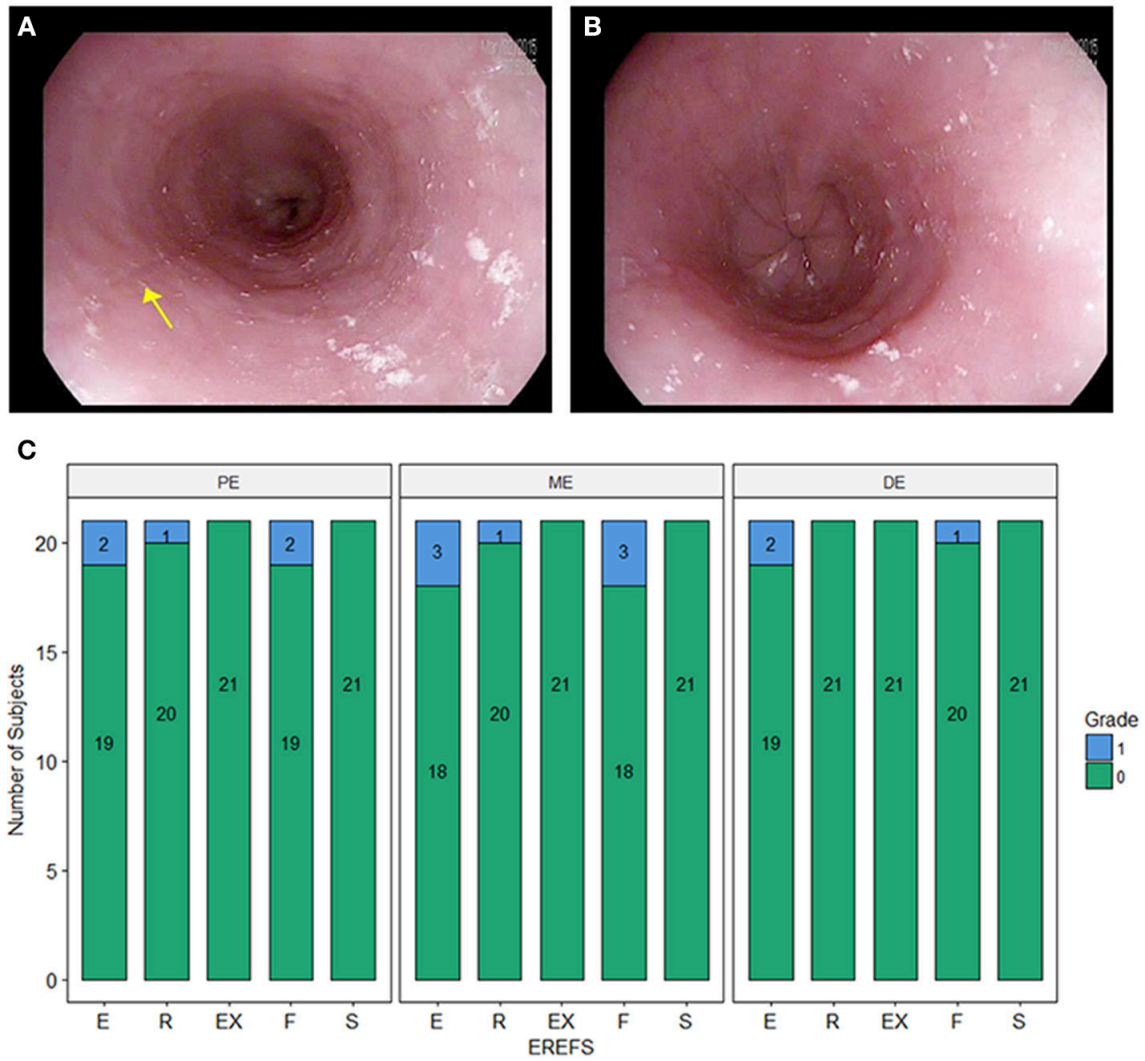
**(A)** Endoscopic picture of the esophagus showing mild longitudinal furrows (e.g., yellow arrow) and mild edema. **(B)** Endoscopic picture of the distal esophagus with mild edema. **(C)** Esophagitis Endoscopic Reference Score (EREFS) distributed over the proximal esophagus (PE), middle esophagus (ME), and distal esophagus (DE). E, edema; R, rings; Ex, exudate; F, furrows; S, strictures.

During EGD, EREFS was documented in all participants. The overall median EREFS was 0 (min = 0, max = 5). No participants had evidence of exudates or strictures. One participant had mild rings (score = 1), three participants had evidence of mild edema (score = 1), and three participants had evidence of mild furrows (score = 1) (Table 2; Figures 1A,B). Figure 1C displays EREFS by PE, ME, and DE. The inflammatory and fibrostenotic scores are reported in Table 2 as the max score across the 3 sites reported in the esophagus. The inflammatory score is the sum of the exudate, edema, and furrows scores (median 0, min = 0, max = 4). The fibrostenotic score is the sum of the rings and stricture scores (median 0, min = 0, max = 3). The total score is calculated from the sum of all 5 findings and in our cohort the median total score was 0 (min = 0, max = 5).

**Table 2:** Endoscopy findings, EREFS.

**Score**	**Total**
Exudates^*^, ***n*** **(%)**	
0	21 (100%)
1	0
2	0
Score, median (min, max)	0 (0, 0)
Rings^*^, ***n*** **(%)**	
0	20 (95%)
1	1 (5%)
2	0
3	0
Score, median (min, max)	0 (0, 1)
Edema^*^, ***n*** **(%)**	
0	18 (86%)
1	3 (14%)
Score, median (min, max)	0 (0, 1)
Furrows^*^, ***n*** **(%)**	
0	18 (86%)
1	3 (14%)
2	0
Score, median (min, max)	0 (0, 1)
Stricture^*^, ***n*** **(%)**	
0	21 (100%)
1	0
Score, median (min, max)	0 (0, 0)
Inflammatory score, median (min, max)^a^	0 (0, 4)
Fibrostenotic score, median (min, max)^b^	0 (0, 3)
Total score, median (IQR)^c^	0 (0, 5)

*EREFS: eosinophilic esophagitis endoscopic reference score*.

* *Maximum score per participant over the three sites*.

a *The inflammatory score is the sum of the exudate, edema, and furrows scores*.

b *The fibrostenotic score is the sum of the rings and stricture scores*.

c *The total score is calculated from the sum of the exudate, edema, furrows, rings, and stricture scores*.

### Histopathology

**Figure 2:**
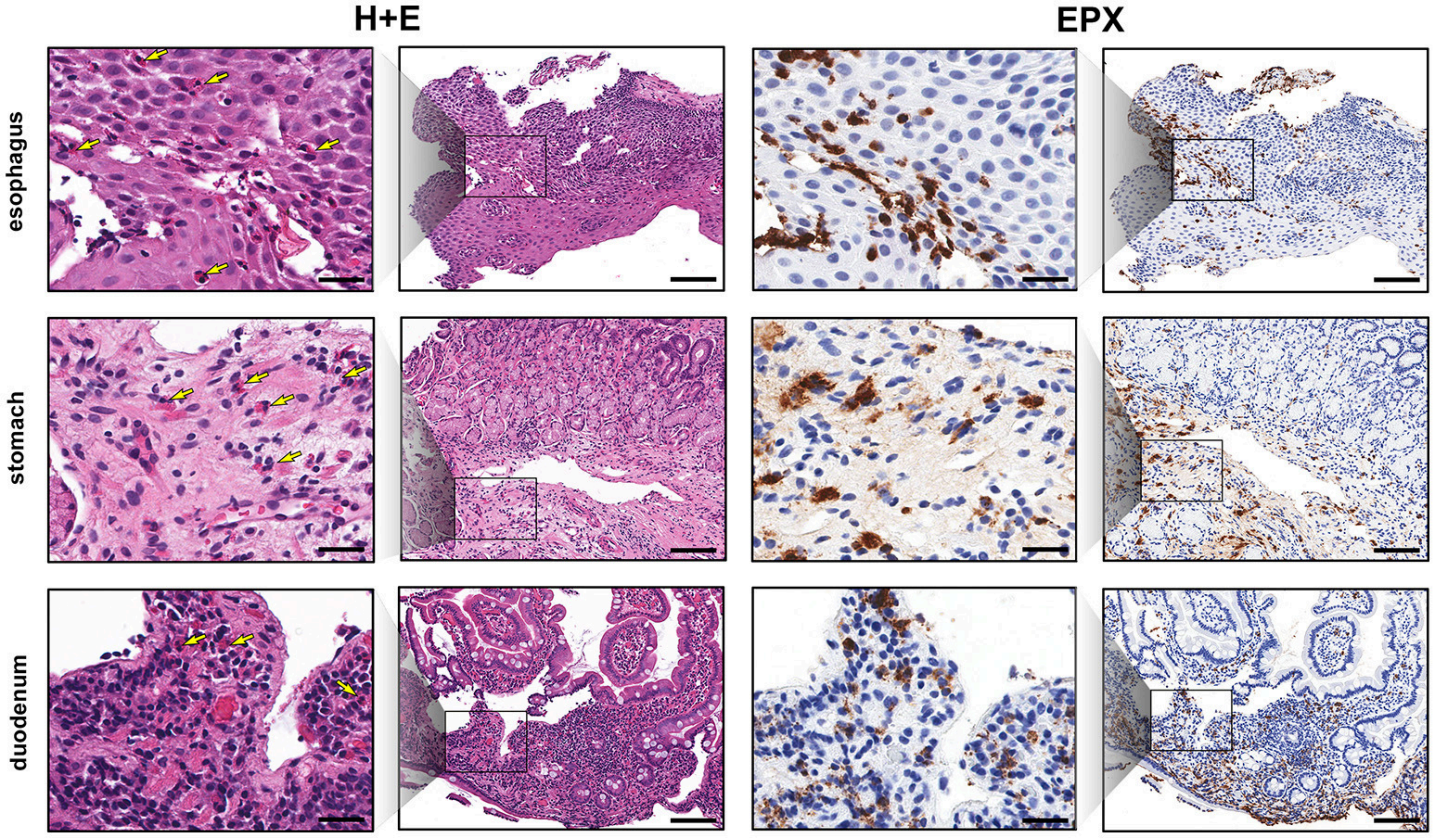
EPX immunohistochemistry highlights gastrointestinal eosinophilic inflammation. Low and high magnification view of serial sections (H&E, columns 1 and 2, and EPX, columns 3 and 4) demonstrating eosinophilia in the esophagus (top row), gastric antrum (middle row) and proximal duodenum (bottom row). Yellow arrows denote eosinophils on H&E stains. Scale bars at high power (columns 1 and 3) and low power (columns 2 and 4) are 25 and 100 microns, respectively.

**Figure 3:**
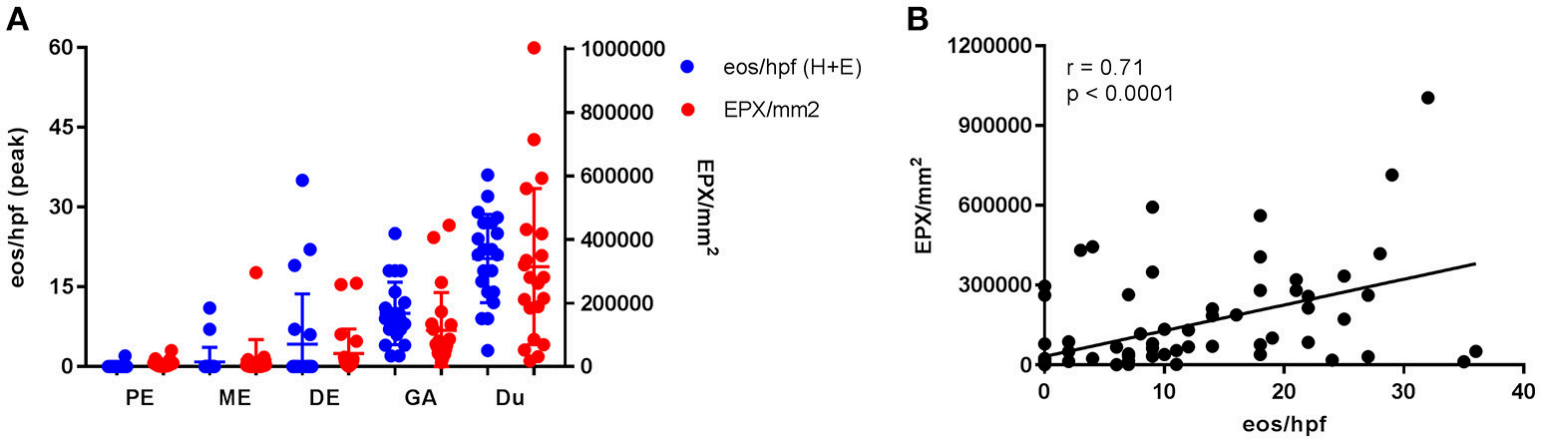
Gastrointestinal eosinophilia is common in adults with IgE-mediated peanut allergy. Eosinophil distribution and EPX deposition **(A)** in the proximal esophagus (PE), middle esophagus (ME), distal esophagus (DE), gastric antrum (GA), and duodenum (Du). Blue circles correspond to the left axis (eos/hpf) and red circles correspond to the right axis (EPX/mm^2^). Eosinophil counts were obtained from H + E stains. When plotted against one another, EPX/mm^2^ correlates strongly with eos/hpf **(B)**.

**Figure 4:**
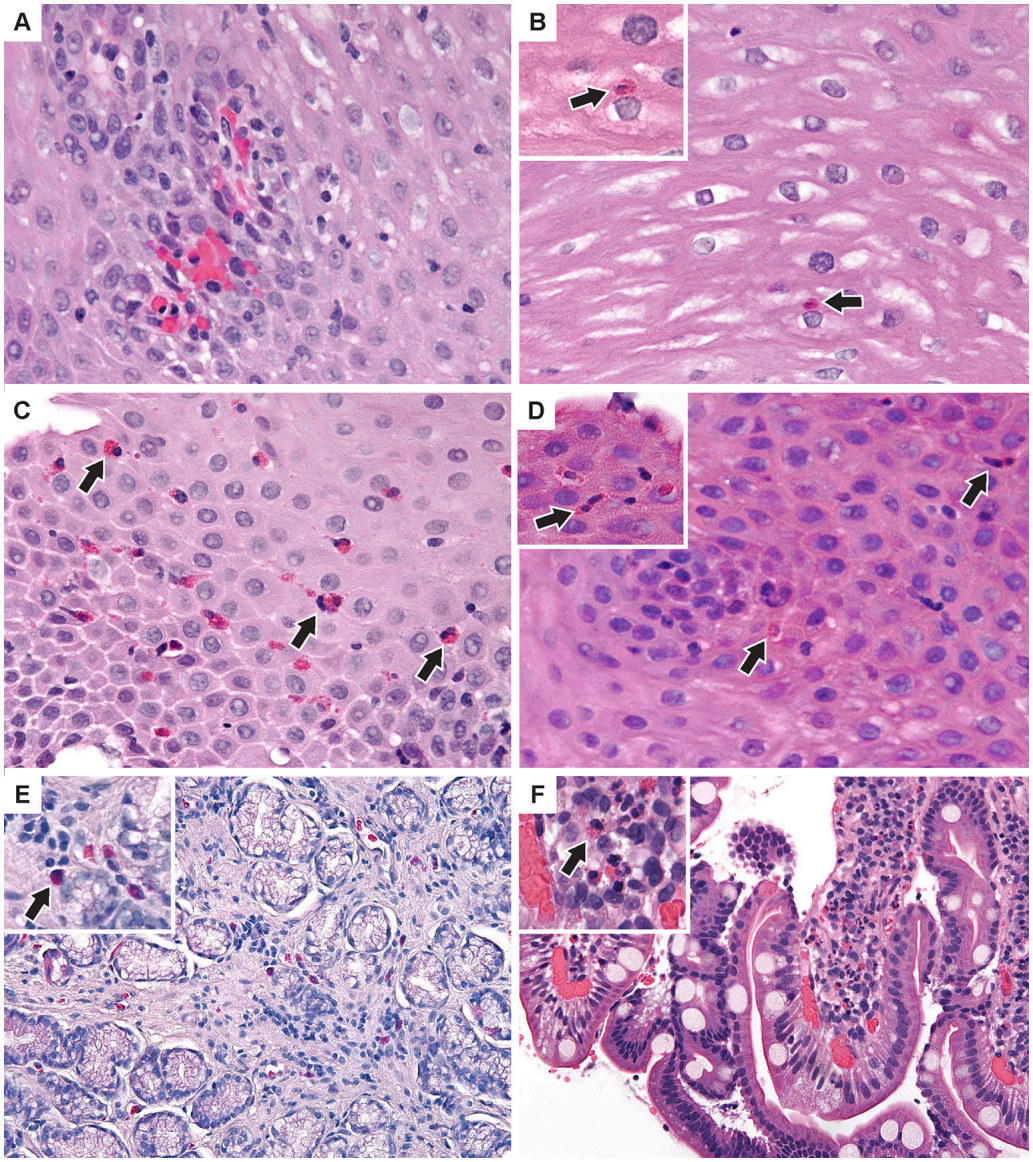
Additional pathologic findings **(A)** Proximal esophagus with increased intraepithelial lymphocytes and congested papillae. Two intraepithelial eos/hpf were seen elsewhere. **(B)** Middle esophagus with normal epithelium and a single intraepithelial eosinophil (inset). **(C)** Distal esophagus with mild basal cell hyperplasia and up to 35 intraepithelial eos/hpf **(D)** Middle esophagus with reactive epithelial nuclei and increased intraepithelial eosinophils (inset) up to 7/hpf. **(E)** Antral stomach mucosa with focal mild non-specific inflammation with lymphocytes and eosinophils (inset). **(F)** Duodenal mucosa with focal lamina propria neutrophils (inset) in one villus. (H&E images × 400). Arrows indicate eosinophils.

**Figure 5:**
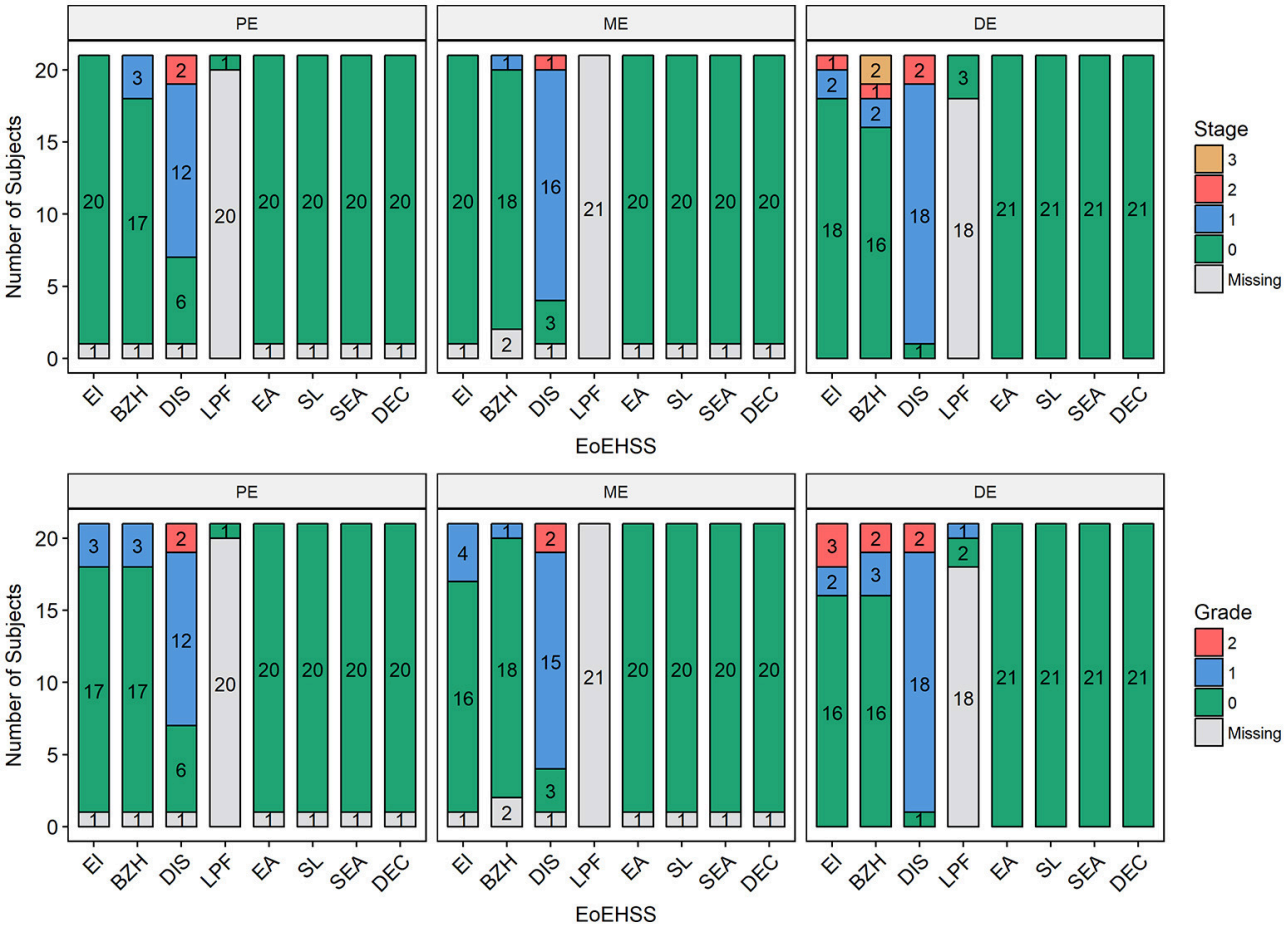
Eosinophilic Esophagitis Histologic Scoring System (EoEHSS). Frequency of histological findings in the proximal esophagus (PE), middle esophagus (ME), or distal esophagus (DE) based on Collins' grading to include eosinophilic inflammation (EI), basal cell hyperplasia (BZH), dilated intercellular spaces (DIS), lamina propria fibrosis (LPF), eosinophilic abscess (EA), eosinophil surface layering (SL), surface epithelial alteration (SEA), and dyskeratotic epithelial cells (DEC). Top panel represents Stage (extent of disease); bottom panel represents Grade (severity of disease).

**Figure 6:**
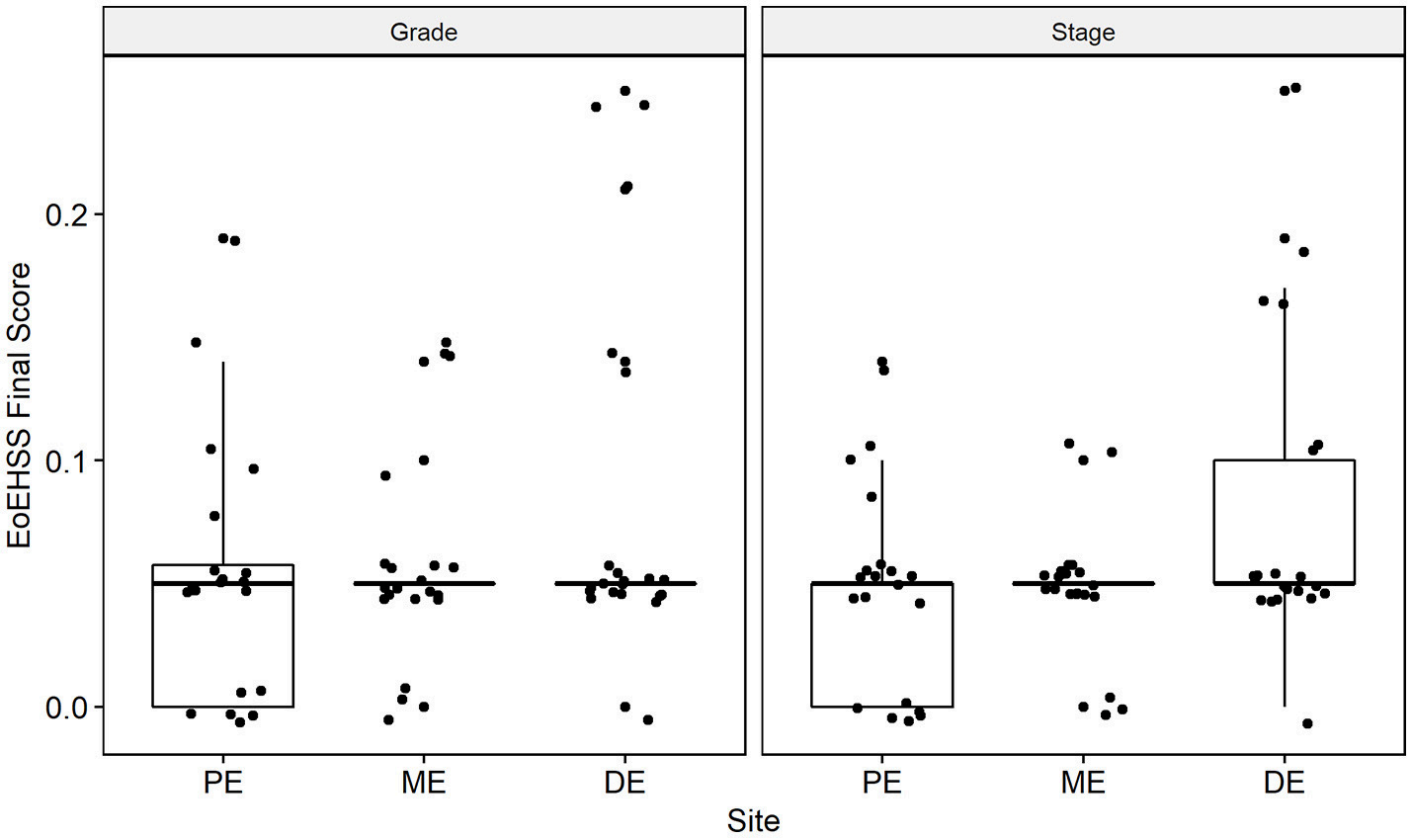
Overall Eosinophil Histologic Scoring System (EoEHSS) at baseline endoscopy. Final score for Grade (left panel) and Stage (right panel) at each site in the esophagus. PE, proximal esophagus; ME, middle esophagus; DE, distal esophagus.

Esophageal eosinophilia (>5 eos/hpf) was present in 24% of participants (Figures 2, 3). Eosinophils were detected by H + E staining in one participant in the PE, two participants in the ME, and five participants in the DE, with three participants reaching the histologic threshold for EoE (≥15 eos/hpf). Further assessment of intact eosinophils and their degranulation products by EPX staining suggest the extent of esophageal eosinophilia may be even greater (Figure 3; Supplementary Figure E2). EPX deposition was greatest in the DE when compared to other esophageal segments and correlated strongly with eos/hpf (*r* = 0.71, *p* < 0.0001). Increased eosinophils were noted in 5 (23.8%) participants in the gastric antrum (>12 eos/hpf) and 6 (28.6%) participants in the duodenum (>26 eos/hpf). Supplementary Table E1 details eosinophil counts for each subject in the various locations of the gastrointestinal tract. While some participants had GE in the stomach and duodenum (29, 39), none met the histologic criteria for eosinophilic gastritis or eosinophilic duodenitis based on H + E stains (40, 41). In addition, none of the biopsies showed intraepithelial eosinophils in the surface or crypt epithelium. Non-eosinophilic histological abnormalities, such as chronic inflammation and intraepithelial lymphocytosis, were found in esophageal biopsies of 3 participants (Figure 4). Histopathologic findings in the PE, ME, and DE using the EoEHSS are summarized in Figure 5; Table 3. Overall, the grade and stage were normal or mildly abnormal in the majority of participants. The lamina propria fibrosis was oftentimes inadequately assessed due to sampling techniques. The median final grade and stage scores were 0.05 across all sites (Figure 6).

**Table 3:** Histology findings: EoEHSS, peak eosinophil counts (PEC) and EPX deposition.

	**Esophageal site**		

	**Proximal**	**Middle**	**Distal**
**Max EoEHSS, Grade (*****n*****, % of cohort)**			
EI	1 (3, 14%)	1 (4, 19%)	2 (3, 14%)
BZH	1 (3, 14%)	1 (1, 5%)	2 (2, 10%)
DIS	2 (2, 10%)	2 (2, 10%)	2 (2, 10%)
LPF	0 (1, 5%)	NA	1 (1, 5%)
EA	0 (20, 95%)	0 (20, 95%)	0 (21, 100%)
SL	0 (20, 95%)	0 (20, 95%)	0 (21, 100%)
SEA	0 (20, 95%)	0 (20, 95%)	0 (21, 100%)
DEC	0 (20, 95%)	0 (20, 95%)	0 (21, 100%)
Non-eosinophilic features, *n* (%)	1 (5%)	0 (0%)	3 (14%)
Peak eosinophil count, median (min, max)	0 (0, 4)	0 (0, 11)	0 (0, 35)
EPX/mm^2^, median (IQR)	8587 (2525-14347)	3776 (540.8-16590)	10356 (4910-26559)

*EI, eosinophilic inflammation; BZH, basal cell hyperplasia; DIS, dilated intercellular spaces; LPF, lamina propria fibrosis; EA, eosinophilic abscess; SL, eosinophil surface layering; SEA, surface epithelial alteration; DE, dyskeratotic epithelial cells; EoEHSS, eosinophilic esophagitis histologic scoring system; EPX, eosinophil peroxidase*.

## Discussion

This is the first study to assess GE by performing endoscopic biopsies in asymptomatic adults with IgE-mediated peanut allergy prior to initiating OIT. We found that 24% of subjects had esophageal eosinophilia at baseline and 14% exceeded the established diagnostic cutoff for EoE, based on PEC. Most of the subjects with esophageal eosinophilia also had additional histologic findings (basal cell hyperplasia, dilated intercellular spaces, and/or lamina propria fibrosis), but these were very mild. The histological abnormalities beyond eosinophilia (as determined by PEC) were assessed by EoEHSS, a newly validated scoring system. EoEHSS objectively and comprehensively evaluates the spectrum of histological changes seen in the esophagus in the setting of EoE. Scoring of eight pathological variables assesses both severity (grade) and extent (stage) of disease. In addition, this system has been shown to be reliable with strong to moderate interobserver agreement among pathologists (37, 42). Based on EoEHSS, despite having baseline eosinophilia in a subset of our cohort, the overall pathology is clearly mild and only a few of the histological abnormalities were noted on endoscopy. Although some of these histopathologic changes resulted in mild endoscopic findings, such as esophageal edema or longitudinal furrows and rings, there were few clinical symptoms and no subjects reported dysphagia. Furthermore, we found that 43% of subjects had increased eosinophils in the gastric or duodenal mucosa, though none met histologic criteria for eosinophilic gastroenteritis based on H + E staining. While there are no current consensus guidelines for cutoff values for eosinophilic gastroenteritis, an average of 30 eos/hpf in 5 hpf is a widely agreed upon histologic threshold for eosinophilic gastritis. Lowichik and Weinberg (39) and Debrosse et al. (29) have both identified the upper limit of normal as 8 eos/hpf in the gastric antrum and 26 eos/hpf in the duodenum in pediatric subjects. Lwin et al. (41) reported higher gastric eosinophil counts (upper limit of normal 11.63 eos/hpf averaged over 5 hpf) in a population of adult and pediatric subjects; however, biopsy samples were obtained from both the corpus and gastric antrum. Although our biopsies were consistently obtained from the gastric antrum, we conservatively defined gastric eosinophilia as >12 eos/hpf for this study.

We found that eosinophil counts were significantly higher based on EPX immunohistochemistry (Supplementary Figure E2), suggesting this may be a more sensitive method for eosinophil detection. However, current reference values (29, 39) and diagnostic cutoffs (40, 41, 43) have been established using routine H + E stains. Moreover, EPX is not a nuclear stain and it assesses both intact eosinophils and degranulation products potentially related to biopsy trauma (38). As a result, there may be an overestimation of eosinophilia in areas of marked degranulation with EPX immunostain. In this cohort, neither eosinophil counts nor EPX deposition corresponded with clinical symptoms as all patients were asymptomatic at the time of endoscopy. Two of the subjects reporting < 3 episodes of abdominal pain per month on the GI symptom questionnaire did have increased EPX deposition in the gastric antrum and one had increased EPX deposition in the duodenum. Larger studies examining the correlation between H + E eosinophil counts and EPX/mm^2^ are needed to established EPX-based diagnostic cutoffs. Using this histologic parameter may allow a more consistent, unbiased and thorough quantification of eosinophilic inflammation.

EoE is a clinicopathologic diagnosis characterized by symptoms of esophageal dysfunction and eosinophilic infiltration of esophageal epithelium (43). While symptoms are required for diagnosis, they may not accurately reflect endoscopic and/or histologic remission following treatment (44). Importantly, the initial presentation in some individuals, particularly adults, is food impaction resulting from esophageal narrowing and fibrostenosis (45) likely caused by progression of undetected chronic inflammation (46). It remains unclear whether the patients with esophageal eosinophilia in this study would eventually progress to develop EoE without intervention, or if the chronic antigen exposure associated with OIT would exacerbate pre-existing pathology.

Despite their status as a defining feature of EGIDs, eosinophils are thought to play important homeostatic roles and are normally present in the gastric and duodenal epithelium; albeit, in lower numbers (47). Increased gastrointestinal eosinophils are also seen in other disease states, such as inflammatory bowel disease (48), gastroesophageal reflux disease (49), celiac disease (50), and connective tissue disorders (51). Interestingly, a common element between EoE and food allergy is epithelial barrier disruption (52). While eosinophils are recognized primarily for their pro-inflammatory potential in disease, they also play important regulatory roles in barrier maintenance through mucus and IgA production, tissue repair, and remodeling (48, 53, 54). For example, the recent description of multiple eosinophil subtypes (55) including Foxp3^+^ eosinophils in EoE (56) provides a more nuanced view of the role eosinophils may play in health and disease. Several studies have examined other cell types which may be upstream of eosinophils in the inflammatory cascade. Indeed, animal models suggest mast cell infiltration of the epithelium precedes eosinophilia (57). Furthermore, basophils have been shown to play an important role in eosinophil recruitment (58). Increases in both cell types have been found in esophageal biopsies obtained from patients with active EoE (59, 60). Interestingly, biologics targeting IL-5 have been largely unsuccessful in alleviating clinical symptoms despite their success in achieving substantial reductions in tissue eosinophilia (61, 62). Taken together, these observations suggest that other cellular targets may also be important for treatment of EGIDs.

The prevalence of GE in asymptomatic individuals with IgE-mediated food allergy is largely unknown and difficult to determine due to absence of non-invasive biomarkers for EGID; notwithstanding, our findings are consistent with those recently reported by Barbosa et al. who performed EGDs in 89 subjects with IgE-mediated milk allergy (63). Thirty-eight percent of subjects in their study had evidence of esophageal eosinophilia. While a majority of those with eosinophilic inflammation had at least some gastrointestinal/non-specific symptoms, almost 30% were asymptomatic. GE does not appear to be as common in patients with other atopic conditions, such as asthma (64), though these studies may be confounded by use of inhaled corticosteroids. Importantly, as many as 67% of patients with EoE report comorbid IgE-mediated food allergy (65). As a result, a patient may have resolution of symptoms but have persistent esophageal eosinophilia despite avoidance of EoE food triggers. Consequently, we may question whether asymptomatic patients with IgE-mediated food allergy and esophageal eosinophilia should receive treatment (i.e., diet modification, steroids, PPI) for “silent” EoE to prevent progression to fibrostenosis. This is a critical issue as delayed diagnosis is associated with stricture formation in symptomatic individuals (66, 67). While Echeverria-Zudaire et al. successfully treated a small cohort of patients undergoing OIT who developed EoE with PPI and possible swallowed steroids, it is unclear whether these participants had underlying gastrointestinal eosinophilia at baseline (27). The present study is an analysis of baseline data from a longitudinal study evaluating the effects of OIT on GE. It has been approved by an IRB and has been closely monitored by a data safety monitoring board of experts in EGID. Due to the absence of clinical symptoms, none of these subjects meet clinicopathologic criteria for diagnosis of EGID, and thus have not received treatment with either PPI or swallowed corticosteroids.

This study has several limitations. First, it is a small, single center study of adult subjects; therefore, it is unclear if similar results would be present in children. In addition, though our population is at much higher risk for EoE, we have applied scoring systems for endoscopic (i.e., EREFS) and histologic (i.e., EoEHSS) findings validated in subjects with EoE (not in IgE-mediated food allergy). Classification of increased gastrointestinal eosinophils distal to the esophagus is also problematic as they are normally present in the stomach and duodenum and reference values are largely derived from pediatric subjects (29, 39). These limitations are balanced by a number of strengths, including a rigorous assessment of symptoms to exclude clinical manifestations of pre-existing EGID, comprehensive endoscopic and histopathologic evaluation, and qualitative and quantitative assessment of GE by EPX immunohistochemistry.

In summary, this study confirms previous speculation that some individuals with IgE-mediated food allergy may have subclinical GE. Endoscopic and histologic findings in the esophagus are mild and are similar to what is seen in EoE that becomes clinically significant. Questions remain regarding the role of eosinophils in IgE-mediated food allergy and the appropriate treatment approach for patients with asymptomatic esophageal eosinophilia. We plan to follow these patients longitudinally to determine the effects of OIT on GE. We will also further examine the role of eosinophils and other cells types during OIT for IgE-mediated food allergy. Surveillance of mucosal responses to OIT may provide a unique window into the pathogenesis of EGIDs.

## Ethics statement

This study was carried out in accordance with the recommendations of ICH/GCP/CFR guidelines by the Stanford IRB with written informed consent from all subjects. All subjects gave written informed consent in accordance with the Declaration of Helsinki. The protocol was approved by the Stanford IRB.

## Author contributions

RSC, KN, and SG designed the research study. BW, KS, AD, NF-B, NK, DT, WZ, BB, MM, and RSC performed the research. BW, MR, AD, EJ, NF-B, NK, NP, SB, SG, and RSC analyzed data. BW and RSC wrote the initial draft of the manuscript. BW, MR, AD, EJ, SB, HK, MT, HM, KN, SG, and RSC provided critical assessments during the revision process leading to the final submitted manuscript. All authors have reviewed and approved the final version of this manuscript.

### Conflict of interest statement

The authors declare that the research was conducted in the absence of any commercial or financial relationships that could be construed as a potential conflict of interest.
