# Supplementary material for: Baseline Gastrointestinal Eosinophilia Is Common in Oral Immunotherapy Subjects With IgE-Mediated Peanut Allergy
Source: Front Immunol. 2018 Nov 22;9:2624. doi: 10.3389/fimmu.2018.02624 (PMC6261984; doi:10.3389/fimmu.2018.02624)
Supplement: Supplementary file 1 [file Data_Sheet_1.docx]

**SUPPLEMENTARY MATERIALS**

**Baseline Gastrointestinal Eosinophilia Is Common in Oral Immunotherapy Subjects with IgE-Mediated Peanut Allergy**

**Benjamin L. Wright, Nielsen Fernandez-Becker, Neeraja Kambham, Natasha Purington, Dana Tupa, Wenming Zhang, Matthew A. Rank, Hirohito Kita, Kelly P. Shim, Bryan J. Bunning, Alfred D. Doyle, Elizabeth A. Jacobsen, Scott D. Boyd, Mindy Tsai, Holden Maecker, Monali Manohar, Stephen J. Galli, Kari C. Nadeau, R. Sharon Chinthrajah**

**FIGURES**

**
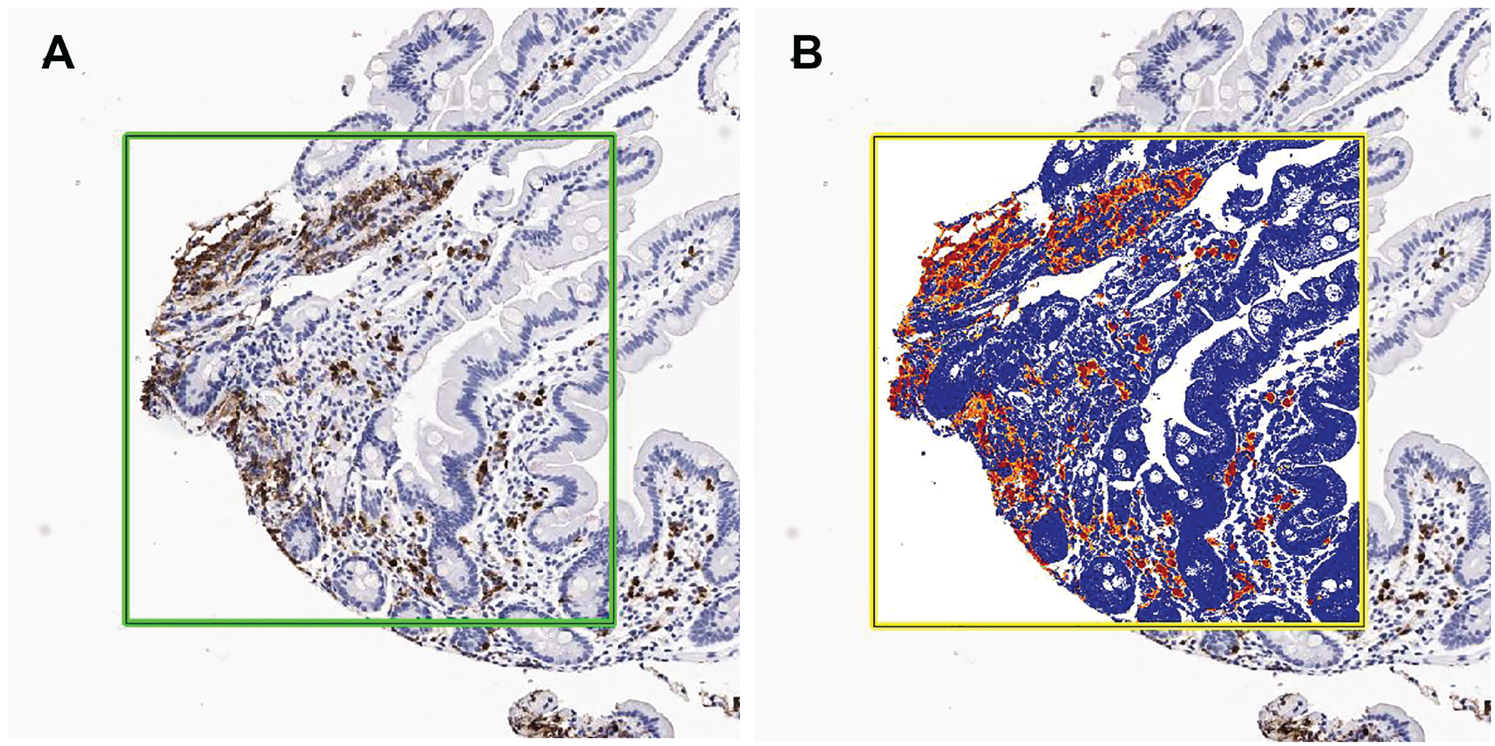
Figure E1.** Sections from a subject with gastric eosinophilia stained for EPX **(A)**. Tissue sections were digitized (Aperio AT Turbo, Leica Biosystems) and EPX deposition was quantified using automated image analysis Aperio ImageScope software (version 11.2.0.780, AperioTechnologies). False color markup **(B)** shows EPX staining strongly positive (red), moderately positive (orange) and weakly positive (yellow). The number of positive red and orange pixels was divided by the epithelial area (mm^2^) analyzed.

**
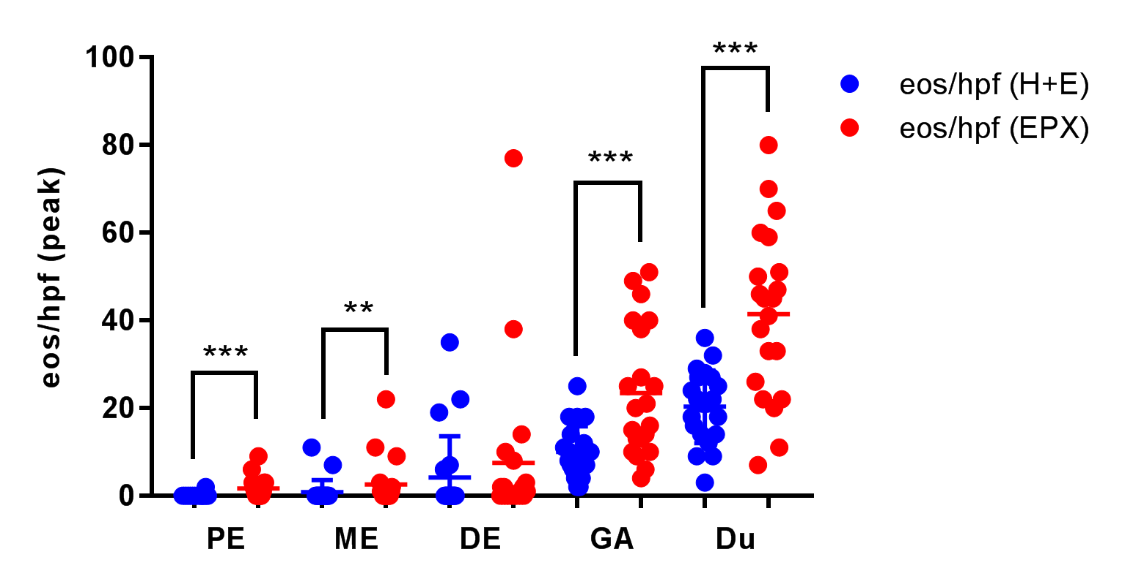
**

**Figure E2**. **Comparison of eosinophil counts by H+E and EPX**. EPX staining by immunohistochemistry facilitated detection of eosinophils resulting in significantly higher eosinophil counts in all segments of the gastrointestinal tract with the exception of the distal esophagus. * p < 0.05, ** p < 0.01, ***, p < 0.001. (PE) proximal esophagus, (ME) middle esophagus, (DE) distal esophagus, (GA) gastric antrum, (Du) duodenum.

SUPPLEMENTARY TABLES

**Table E1. Detailed participant findings on histology**

| Subject No. | Proximal esophagus  (eos/hpf) | Mid esophagus  (eos/hpf) | Distal esophagus  (eos/hpf) | Gastric antrum  (eos/hpf) | Duodenum  (eos/hpf) |
| --- | --- | --- | --- | --- | --- |
| 1 | 0 | 0 | **19** | 6 | 18 |
| 2 | 0 | 0 | 0 | **14** | **29** |
| 3 | 0 | 0 | 0 | 9 | **27** |
| 4 | 0 | 0 | 0 | 4 | 14 |
| 5 | NA | 0 | **6** | **25** | 9 |
| 6 | 1 | **11** | **7** | 10 | **32** |
| 7 | 0 | 0 | 0 | 2 | 9 |
| 8 | 1 | 1 | **35** | **18** | **36** |
| 9 | 0 | 0 | 0 | 12 | 21 |
| 10 | 4 | **7** | **22** | 11 | **27** |
| 11 | 0 | 0 | 0 | 7 | 14 |
| 12 | 0 | 0 | 0 | **18** | 12 |
| 13 | 0 | 0 | 0 | 10 | 25 |
| 14 | 0 | NA | 0 | 9 | 22 |
| 15 | 0 | 2 | 0 | **18** | 22 |
| 16 | 0 | 0 | 0 | 9 | 21 |
| 17 | 0 | 0 | 0 | 7 | 24 |
| 18 | 0 | 0 | 0 | 2 | 3 |
| 19 | 0 | 0 | 0 | 8 | **28** |
| 20 | 0 | 0 | 0 | 4 | 18 |
| 21 | 0 | 0 | 0 | 7 | 16 |

Eosinophil counts per high powered field (eos/hpf) with eosinophils > 5 eos/hpf (esophagus), > 12 eos/hpf (stomach), and > 26 eos/hpf (duodenum) bolded.

NA: tissue not available

**Table E2: Food allergy history and concurrent medication use**

| Subject No. | Other food allergies and avoidance | PPI/ H2B Use | ICS Use |
| --- | --- | --- | --- |
| 1 | No | No | No |
| 2 | Milk | No | No |
| 3 | Hazelnut | No | No |
| 4 | No | No | No |
| 5 | No | No | No |
| 6 | Walnut, Almond, Pecan, Soy | No | No |
| 7 | No | No | No |
| 8 | No | No | No |
| 9 | Walnut, Cashew, Almond, Pecan, Hazelnut, Sesame, Shellfish | No | Yes |
| 10 | Walnut, Cashew, Almond, Pecan, Hazelnut, Egg, Milk, Fish | No | Yes |
| 11 | Egg | No | No |
| 12 | No | No | No |
| 13 | No | No | No |
| 14 | Walnut, Cashew, Almond, Pecan, Hazelnut | No | No |
| 15 | Egg | No | No |
| 16 | Walnut, Cashew, Fish | No | No |
| 17 | No | No | No |
| 18 | No | No | No |
| 19 | No | No | No |
| 20 | Walnut, Cashew, Almond, Pecan, Hazelnut, Egg, Milk | No | No |
| 21 | No | No | No |

Co-existing food allergies and regular, daily, concomitant medication use is noted for each participant: proton pump inhibitor (PPI); histamine receptor 2 blocker (H2B); inhaled corticosteroids (ICS).

GASTROINTESTINAL SYMPTOM QUESTIONNAIRE
